# Characterization of Magnesium-Polylactic Acid Films Casted on Different Substrates and Doped with Diverse Amounts of CTAB

**DOI:** 10.3390/molecules26164811

**Published:** 2021-08-09

**Authors:** Margarita Hierro-Oliva, Verónica Luque-Agudo, Amparo M. Gallardo-Moreno, M. Luisa González-Martín

**Affiliations:** 1 Department of Applied Physics, Faculty of Science, University of Extremadura, 06006 Badajoz, Spain; margahierro@unex.es (M.H.-O.); vluque@unex.es (V.L.-A.); amparogm@unex.es (A.M.G.-M.); 2 Networking Research Center on Bioengineering, Biomaterials and Nanomedicine (CIBER-BBN), 06006 Badajoz, Spain; 3 University Institute of Extremadura Sanity Research (INUBE), 06006 Badajoz, Spain

**Keywords:** polylactic acid, surfactants, magnesium, surface characterization

## Abstract

Polylactic acid (PLA) is a good candidate for the manufacture of polymeric biodegradable biomaterials. The inclusion of metallic particles and surfactants solves its mechanical limitations and improves its wettability, respectively. In this work, cetyltrimethylammonium bromide (CTAB) and magnesium particles have been incorporated into PLA films to evaluate the changes produced in the polymeric matrix cast on glass and silicone substrates. For this purpose, the surface of the films has been characterized by means of contact angle measurements and ToF-SIMS. Depth profiles and SEM images of the cross sections of the films have also been obtained to study their morphology. The results show that the CTAB in the polymer matrix with and without magnesium improves the wettability of the films, making them more suitable for cell adhesion. The higher the hydrophilicity, the higher the surfactant concentration. The depth profiles show, for the first time, that, depending on the surfactant concentration and the presence of Mg, there is a layer-like distribution near the surface where, in addition to the CTAB + PLA mixture, a surfactant exclusion zone can be seen. This new structure could be relevant in in vitro/in vivo situations when the degradation processes remove the film components in a sequential form.

## 1. Introduction

Polylactic acid (PLA) is undoubtedly one of the most valuable, biodegradable, bioabsorbable, and sustainable polymers. Its use mainly covers the manufacture of biomedical devices, but also extends to different food, cosmetic, and pharmaceutical packaging applications [1]. Its interesting properties of non-toxicity and biocompatibility have prompted research to improve some of its other characteristics that appear as shortcomings in order to broaden its field of application. In particular, its mechanical behavior is very limited [2]. Its low ductility is one of its main drawbacks as a packaging material. On the other hand, even under low levels of stress, PLA tends to deform, permanently causing the loosening of the biodegradable fixation, and its low mechanical strength makes it unfeasible for a PLA orthopedic implant to maintain adequate performance until the bone grows sufficiently to restore its functionality [2].

Different strategies have been pursued to adapt PLA to the requirements necessary for its intended use [3]. A common methodology to endow the material with new properties is the inclusion within the polymeric matrix of dispersed particles or fibers. Cellulose and eucalyptus microfibers are added to improve the mechanical properties and barrier properties of PLA for food packaging applications [4,5,6]. Hydroxyapatite or magnesium phosphate fibers are included in PLA for the electrospinning fabrication of biomedical devices [7,8].

However, one of the most promising alternatives to improve the mechanical behaviour of PLA is the addition of magnesium particles to the polymer matrix [9,10]. Magnesium is a biocompatible and biodegradable metal. Its use, pure or in alloy, is intensively investigated for the fabrication of implants [11,12]. However, the low corrosion resistance and undesirable hydrogen production of this metal has led researchers to consider the inclusion of Mg microparticles in a PLA matrix as an alternative composite material. The biodegradability of magnesium appears as a very complementary property to the biodegradability of PLA. This synergy makes this composite very attractive as it meets the mechanical properties suitable for use in orthopedic devices and allows the implant to be fully reabsorbed by the body [13].

The first contact between an implant and the physiological environment is through the surfaces. Regarding biocompatibility, wettability is a determining characteristic of the material surface [14]. Arima and Iwata posited that surfaces whose water contact angle is around 40–60° are suitable for HeLa cells adhesion [15], and in general, moderately hydrophilic surfaces have been shown to be the most suitable for cell adhesion, spreading, and growth [14]. From this perspective, PLA needs improvements as its surface is hydrophobic. The inclusion of surfactants within the polymer matrix is one methodology followed to decrease the hydrophobicity of PLA. Zhang and Severtson studied the distribution of sodium alkyl sulphate surfactants in a polymer film and found that due to the enrichment of the interface with surfactant, the polymer contact angle changed from ~110° to ~15°, depending on the concentration and the surfactant [16]. Kiss et al. using Pluronic surfactants [17,18] found differences in water contact angle between 40° and 10° for PLA films without and with surfactants. Similar results were found by many other researchers working with different surfactants, such as Fukuoka et al. [19] or Gifu et al. [20] among others [17,21]. However, Gromer et al. [22], studying the surfactant distribution in films made from polymeric colloids, have shown that there are a large number of factors affecting the surfactant content along the film thickness, such as the type of surfactant and its initial concentration, the inorganic particles present, the size, shape, and thickness of the film, the aging conditions, and even the substrate on which the film is poured.

On this basis, the objective of this research is to evaluate the surface and internal changes of PLA composite films with magnesium particles due to the inclusion of a surfactant in their preparation. The films will be prepared by solvent casting, without the presence of water, using two substrates with different hydrophobicity for casting that can induce changes in the surface properties of the material [19]. The surfactant will be cetyltrimethylammonium bromide (CTAB). This cationic surfactant is relatively safe and frequently used in disinfection and cleaning procedures because of its antimicrobial properties [23,24], in addition to the fact that even a trace amount of surfactant provides an effective strategy to reduce biofilm formation [25].

Wettability and compositional distribution will be the parameters analyzed. Therefore, water and diiodomethane contact angles will be measured on the films to evaluate their hydrophobicity and surface free energy. Likewise, the ToF-SIMS technique will be used to acquire the mass spectra at the surface and as a function of the film depth, for each one of the samples under study, in order to ascertain the distribution of the surfactant within the matrix as a function of the surfactant concentration, presence of magnesium, and substrates used in the manufacture.

## 2. Materials and Methods

### 2.1. Films Preparation

Glass disks samples were immersed in chromic acid for 15 min and then rinsed with distilled water. Silicone wafers were rinsed with absolute ethanol and dried with a flow of N_2_. They were kept in the desiccator until use.

Polylactic acid (PLA) particles (PLA2003D, with d-isomer content of 4.25%, purchased from NatureWorks LLC, Blair, NE, USA) were dissolved in chloroform (Sigma-Aldrich, Merck, Darmstadt, Germany) (5% *w*/*v*) using a rotator stirrer (JP Selecta, Barcelona, Spain). For preparation of films containing magnesium, magnesium particles (≤50 µm, Nitroparis, Castellón, Spain) were added (3% *w*/*w*) to the solution and stirred until complete homogenization. To determine the concentration of magnesium to be included, previous adhesion and bacterial viability tests were carried out, choosing the 3% (*w*/*w*) concentration because it causes bacterial damage but without reaching 100% mortality. Once the polymer solutions were prepared, varying amounts of cetyltrimethylammonium bromide (CTAB, Sigma-Aldrich, Merck, Darmstadt, Germany) (0.5, 1, 5, and 10% *w*/*w*) were added and stirred until completely dissolved. These CTAB concentrations were chosen to sweep a wide range around the MIC.

The nomenclature system of the films is as follows: A-PLA-x/y, where A is S for films casted on silicone and G for films casted on glass; *x* represents the magnesium concentration and can be 0 or 3% (*w*/*w*) and *y* represents the CTAB concentration, and can be 0, 0.5, 1, 5, and 10% (*w*/*w*).

Briefly, 1 mL or 2 mL of each solution was cast on silicone wafers or glass disks (respectively). The volumes of solution to be deposited depend on the area of the substrate and the thickness of the film to be obtained. Thus, since the diameter of the samples is different, it is necessary to deposit 2 mL of PLA solution on the glass to obtain a thickness equivalent to that of the samples prepared on silicone. Samples were left to dry first at room temperature for 24 h and later in an oven at 70 °C for 24 h, to completely remove any remaining solvent [26]. After that, the films thus prepared were peeled off the silicone wafers and glass disks. Surface characterization was carried out on the side in contact with the substrate. Complementary measurements were also made on the side in contact with the air to check if both sides behaved similarly.

### 2.2. Contact Angle Measurement

Static deionized water (Milli-Q Integral 5 System, Merck, Darmstadt, Germany) and diiodomethane (Fluka, Thermo Fisher Scientific, Waltham, MA, USA) contact angle measurements were carried out with a Drop Shape Analyzer-DSA100E system (Krüss, Hamburg, Germany) by the sessile drop method. Drops were photographed at 15 s after deposition. The results of contact angles, namely averaged values of at least three independent films, were analyzed for each of the conditions studied and two drops of each liquid were deposited per film.

### 2.3. Surface Structure Characterization

Cross sections of the films were obtained with the aid of a surgical knife. Microstructural analyses of cross-sectioned samples were performed by scanning electron microscopy (SEM, Hitachi S-4800, Chiyoda, Tokyo, Japan). The operation was performed under high vacuum conditions, with gold coating of the surface of the section exposed. Images were taken in the secondary electron (SE) mode.

### 2.4. Surface and Depth Profile Chemical Composition

Time of flight secondary ion mass spectrometry (ToF-SIMS) analyses of samples were performed with a Tof-SIMS^5^ (ION TOF, Münster, Germany) using a Bi_3_^2+^ as primary gun, which operated at 25 keV. The total ion dose used to acquire each spectrum was above 10^12^ ions/cm^2^. Negative spectra were recorded, and a pulsed low energy electron flood gun was used for charge neutralization. For samples containing Mg, positive spectra were also collected to see the evolution of the Mg^2+^ ion. The depth profiles were performed using a Bi_3_^2+^ beam for analysis and an Ar^+^ cluster (size of the cluster 1550 atoms) as a sputtering source, which operated at 10 keV energy. The total ion dose was above 10^12^ ions/cm^2^ per spectrum also for the depth profiles. The spot size for the surface spectra was 250 μm × 250 μm and for the depth profiles was 300 μm × 300 μm of sputter area and 50 μm × 50 μm of analysis area.

The depth of the crater made in the depth profile was measured by optical profilometry, using a 3D Optical Surface Metrology System Leica DCM8 (Leica, Wetzlar, Germany).

## 3. Results and Discussion

### 3.1. Surface Analysis of the Films

The presence of varying amounts of CTAB and magnesium particles and the use of two types of substrates (one hydrophobic and another hydrophilic) to cast the PLA films can induce changes in the surface properties of the material [19]. Moreover, after manufacturing the films, we observed, especially for the films with higher CTAB content, a greater accumulation of CTAB at the edges than in the center, and a better homogeneity in this area. For this reason, we decided to carry out all the measurements in the center of the films and not at the edges because, in this latter region, the evaporation is faster and the particles may pack up sooner [27], keeping traces of the solvent occluded.

**Figure 1:**
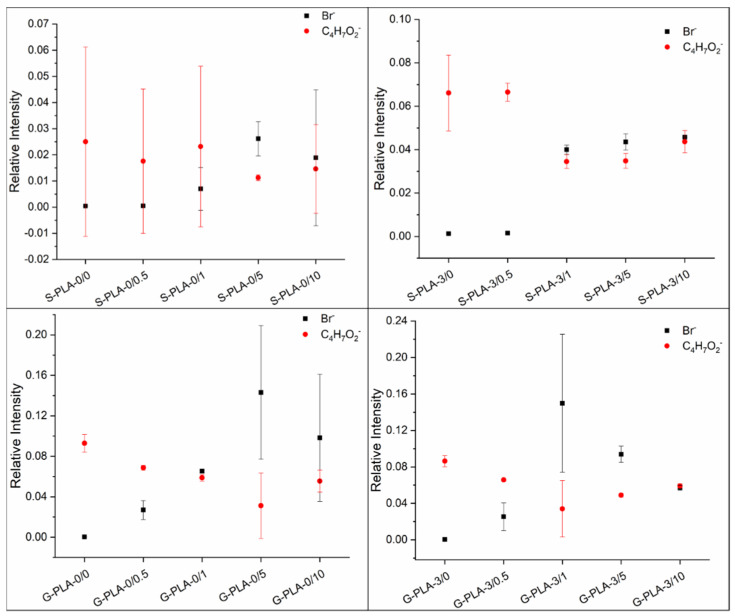
Relative intensities of Br^−^ and C_4_H_7_O_2_^−^ ions as a function of sample type for PLA films containing CTAB and Mg casted on silicone and glass.

The surface composition of the films was evaluated by analyzing the ToF-SIMS surface mass spectra. Figure 1 shows the relative intensities of the characteristic ions of the surfactant and the polymer as a function of sample type. The bromide ion (Br^−^) was chosen as a representative fragment to determine the CTAB content, while the C_4_H_7_O_2_^−^ fragment was selected for PLA. Both the surfactant and the polymer have hydrocarbon moieties in their structures, but the one considered for the analysis is unequivocally assigned to PLA and not to CTAB as it contains oxygen and no nitrogen.

In general, the bromide ion is poorly detected on the surface of films prepared with low CTAB concentrations and when the surfactant concentration increases this trend is reversed and the presence of surfactant becomes noticeable on the surface. This observation also seems to be dependent on the presence of Mg inside the film. In the films without Mg (S-PLA-0/x and G-PLA-0/x), CTAB starts to be relevant on the surface at concentrations of to 5% (*w*/*w*) and above, while in Mg-containing films CTAB starts to be present on the surface from a concentration of 1% (*w*/*w*).

The side of the film exposed to air during the drying process has also been analyzed and, although the trend is similar (Supplementary Materials), no changes as a function of CTAB content were observed in the values of the contact angles measured on that side (unpublished data). The study of the wettability of the samples as a function of the contact angle values was carried out on the side of the film in contact with the substrate (Table 1).

The data shown in Table 1 indicate that there are significant differences between the water (θ_W_) contact angles depending on the substrate on which the samples are cast. The contact angles for water are always higher for samples deposited on silicone than on glass. This may be due to the difference in crystallinity between the two types of films. Li et al. [28] related an increase in the hydrophobicity of PLA to an increase in its crystallinity, because the methyl groups of the polymer chain were more exposed towards the surface. This orientation can occur naturally/spontaneously or can be mechanically induced, as demonstrated by these authors. In our case, it seems that, in S-PLA-0/0 films, these hydrophobic methyl groups tend to be oriented towards the silicone, also hydrophobic, while in G-PLA-0/0 films, due to the hydrophilic character of the substrate, these groups would be oriented towards the bulk. The high crystallinity of the films casted on silicone was already shown in a previous work of our group [26], in which the peaks corresponding to the α crystalline phase appeared to be very well defined. In this phase, the methyl groups were oriented outwards from the polymeric chain, which would corroborate the hydrophobicity of these samples and support the hypothesis stated above.

The differences between the contact angles of the samples casted on both substrates are even more significant when Mg is present in the film: water contact angle values are on average 30% lower for glass samples than for silicone samples. In our previous work [26], we also observed small differences in crystallinity when the films were doped with Mg particles (10% *w*/*w* in that case). Although PLA crystallized in the α phase, we did find small displacements of the peaks in the diffractogram, due to a distortion in the polymer chain caused by the presence of the Mg particles. This distortion affects the packing and the spacing between chains, likely being responsible for the differences found in the contact angles.

Regarding CTAB-containing films, it has already been described that a higher presence of CTAB in polymeric samples increases the surface hydrophilicity [17]. This increase in wettability with the surfactant accumulation on the film surface was already observed by Gyulai et al. [21] using XPS analysis and working with different surfactant concentrations. Their study concluded that as the surface became richer in surfactant, it became more hydrophilic. In our case, the water contact angles have a common trend for both types of substrates and their values decrease when there is a high concentration of CTAB on the surface of the film (Figure 1 and Table 1). For both substrates, on films containing 0.5% (*w*/*w*) of CTAB, the average water contact angle does not change, so small amounts of CTAB are not enough to modify the hydrophobic behavior of the polymer. This is in agreement with data inferred from ToF-SIMS surface mass spectra (Figure 1), where no significant amount of CTAB on the surface is detected at low surfactant concentration. In the case of higher CTAB concentrations, two factors could contribute to the significant changes in hydrophobicity. Firstly, it seems that the CTAB presence may decrease the crystallinity of PLA [10], and secondly, the polar head of CTAB (containing the Br^−^ ion and the quaternary ammonium fragment) has hydrophilic character, so that, as the surfactant is already on the outermost face of the film (Figure 1), the hydrophobicity of the samples decreases. When Mg is present in the film, CTAB also hydrophilizes the surfaces, similarly to what happens in Mg-free films, but the differences are higher, probably because CTAB affects how the polymer chains are packed together with the Mg particles.

It is also noteworthy that the film surface seems to become saturated with CTAB from 5% (*w*/*w*) since samples containing 5% and 10% behave similarly within standard deviations. In those cases, the saturation in the film surfaces may provoke the displacement of the excess of surfactant both towards the edges and outwards. The transport of matter by horizontal diffusion has already been described by other authors [29,30].

Regarding the vertical distribution of the surfactant in the film, its molecular structure must be taken into account. Gromer et al. [22] stated that hydrophobic surfactants are distributed homogeneously throughout the film, while hydrophilic ones tend to accumulate at the air and substrate sides, leaving the bulk impoverished. Moreover, Zhang and Severtson [16] explained the differences in accumulation between the film–substrate and the film–air interfaces, only in the case of surfactants dissolved in water. In that case, evaporation was faster at the film–air interface (because there was a direct contact with the atmosphere) than at the film–substrate interface. This was the reason for the higher surfactant accumulation at the film–air interface. In our case, CTAB is dissolved in chloroform where, although it is soluble, it is not as soluble as in water. This could explain the predominance of the interaction between the surfactant and the substrate over the evaporation rate of the chloroform at the film–air interface, leading to a higher accumulation of CTAB on the side of the film in contact with the substrate compared to the side exposed to the air. In addition, the compatibility of the system under study must be considered. In this range of high CTAB concentrations, according to our results (Figure 1) and in agreement with Mallégol et al. [31], it seems that during drying an excess of surfactant accumulates at the film–substrate interface [32,33].

Additionally, the contact angles of diiodomethane on these samples were measured (Table 1) in order to calculate the surface tension and its components, as summarized in Table 2. As mentioned above, the diiodomethane (θ_D_) contact angles depend on the substrate on which the samples are casted, being higher for samples deposited on silicone than on glass. Moreover, in Mg-containing films, diiodomethane contact angles are on average 20% lower for glass samples than for silicone samples.

The results obtained are in agreement with the hydrophobicity and chemical composition of the surface of the films: changes in both θ_W_ and θ_D_ also imply changes in the surface tension of the solid (γ_S_) and especially in the polar component (γ^p^).

**Figure 2:**
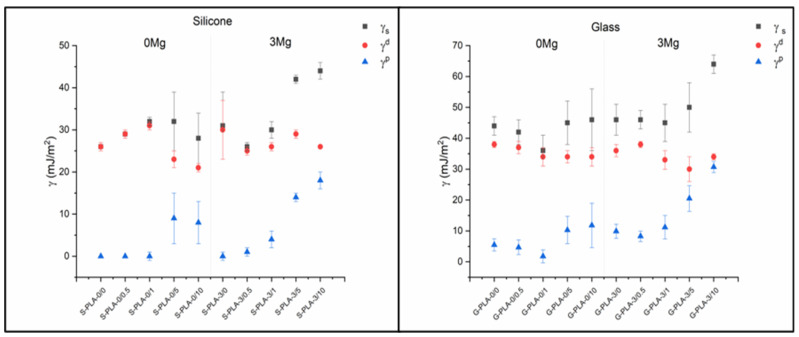
Surface tension (γ_S_) and dispersive (γ^d^) and polar (γ^p^) components calculated using the Fowkes model.

There is also a clear difference between S-PLA-0/0-0.5 and G-PLA-0/0-0.5 films (Table 2). Samples deposited on glass with small amounts of CTAB have a low but non-zero γ^p^ value, which infers a certain polarity to the surface, while those prepared on silicon are non-polar (γ^p^ = 0 mJ/m^2^). For both substrates, there is a clear increase in the γ^p^ component as the amount of CTAB in the samples increases (Figure 2). For films non-containing Mg, the inclusion of CTAB at concentrations above 5% (*w*/*w*) induces a significant increase in the polar component. The same behavior is also observed for the Mg-containing films, but at CTAB concentrations of 1% (*w*/*w*). Moreover, the increase of the polar component is much higher. In the dispersive component, it is noteworthy that, in films deposited on silicone and not containing magnesium, no significant change is observed up to CTAB concentrations higher than 1% (*w*/*w*). This trend is also valid for samples deposited on glass, with the difference that the γ^d^ component begins to decrease for concentrations higher than 0.5% (*w*/*w*). For magnesium-containing samples, deposited on both silicon and glass, the dispersive component shows practically no change as a function of the amount of CTAB, taking into account the uncertainties associated with each value. For the surface tension, taking into account the uncertainties, no significant changes are observed as a function of the CTAB concentration, except in the case of films containing magnesium and with a very high surfactant content (5 and 10% *w*/*w*).

### 3.2. Internal Analysis of Films

In order to check the internal distribution of components inside the casted films, the samples were analyzed by ToF-SIMS depth profiles and SEM cross-section images.

**Figure 3:**
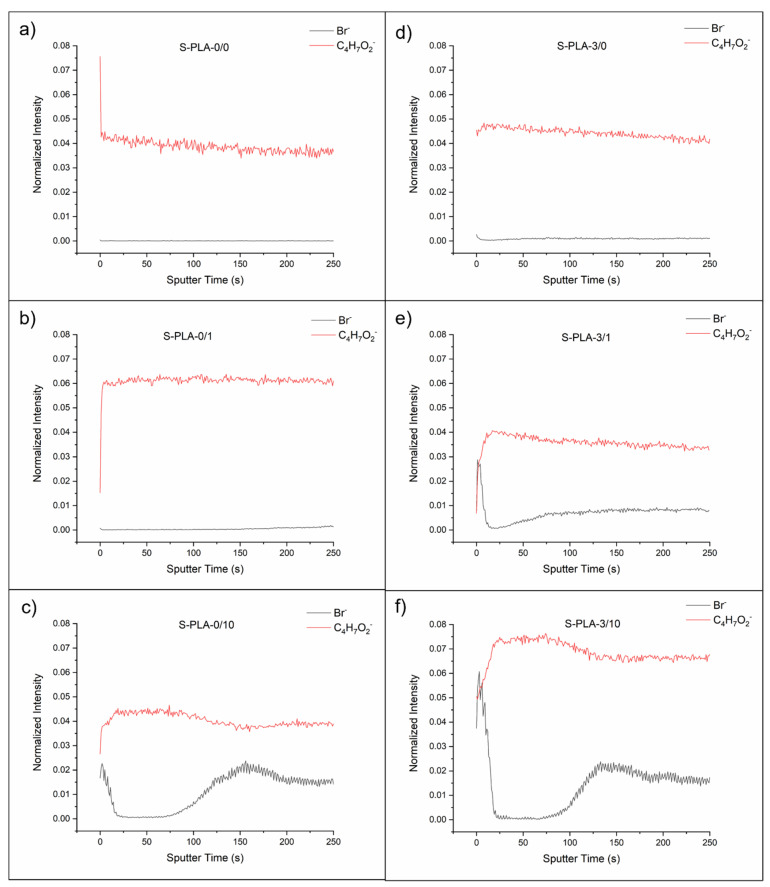
Depth profiles of S-PLA-0/0 (**a**), S-PLA-0/1 (**b**), S-PLA-0/10 (**c**), S-PLA-3/0 (**d**), S-PLA-3/1 (**e**) and S-PLA-3/10 (**f**) films.

Figure 3 shows the depth profiles of the films casted on silicone with and without magnesium and without CTAB, with 1 and 10% (*w*/*w*) of CTAB. Depth profiles of the films casted on glass are analogous to S-PLA films (see Supplementary Materials).

In films without Mg (S-PLA-0/x), the presence of CTAB on the surface is only notorious at 10% *w*/*w* concentration (Figure 3c). Interestingly, at this concentration, just below this outer surface layer, CTAB is not found (between 20 and 75 s of sputtering, approximately), and then surfactant is again detected. From this information it can be derived that at 10% *w*/*w*, there is a first layer of polymer, with a significant presence of CTAB, immediately followed by a layer of polymer and, after 75 s of sputtering, a third layer in which both PLA and surfactant are continuously blended.

In the case of films with Mg (S-PLA-3/x), this layered structure is already observed for concentrations of 1% *w*/*w*, the first layer is somewhat diffuse and both components are blended (Figure 3e). The second layer is mainly composed of polymer and, at a depth located at about 30 s of sputtering, the third layer of PLA and CTAB mixed is found again. At higher concentrations, the layered structure is maintained (Figure 3f).

In order to confirm the presence of magnesium inside the film, the depth profiles were recorded in positive mode to track the Mg^2+^ cation (unpublished data), because in negative mode MgO^−^ anion induces low signals. Moreover, as the applied ionization energy cannot be too high to extract the organic ions from the polymer–surfactant matrix, the amount of Mg^2+^ cations extracted in positive mode is enough to confirm their presence but is still low compared to the rest of the ions detected.

The depth profiles of the films were also recorded starting from the side exposed to air (see Supplementary Materials). In this case, as previously described in the surface analysis of films, it was observed that the accumulation of CTAB on the surface (higher the higher the surfactant concentration) was smaller than the accumulation on the film–substrate interface, which was related to the similar contact angles measured on that side. The layer structure observed on the surface in contact with the substrate is not so clearly defined in this case, with the outermost layer already being a mixture of CTAB and PLA.

Even though the layer-like structure inside the films has not been described before, data are partially supported by the research of Zhang and Severtson [16]. These authors showed that anionic surfactants tended to accumulate on the film surfaces, while non-ionic surfactants were homogeneously distributed throughout the film. Although the surfactant used in this work is cationic, electrical interactions must also be responsible for its increased accumulation on the surface, similar to that observed for anionic surfactants. This idea is also reinforced by the fact that the presence of Mg causes the accumulation of CTAB on the surface to be observed at lower surfactant concentrations, since the Mg^2+^ ions, dispersed in the polymeric matrix, must favor electrical repulsions with the CTAB molecules.

**Figure 4:**
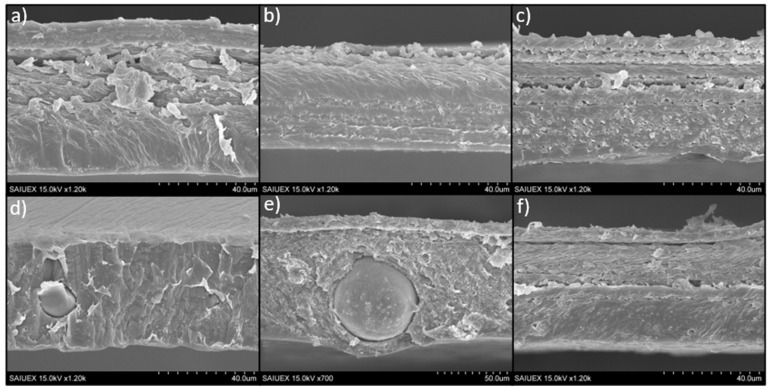
Cross-section images of S-PLA-0,0 (**a**), S-PLA-0,1 (**b**), S-PLA-0,10 (**c**), S-PLA-3,0 (**d**), S-PLA-3,1 (**e**) and S-PLA-3/10 (**f**) films.

The morphology of the cross sections of the films was revealed by SEM images, as shown in Figure 4. In general, images show in some cases certain laminar structure of the polymeric matrix, which can be related to the sequential drying of the film (giving different arrangement of the polymer chains) or even to the mechanical forces exerted on the films during the peeling off from the silicone disks. This laminar structure is hard to relate to the different PLA-CTAB layers described previously in the depth profiles because the thickness of the ToF-SIMS craters, measured by optical profilometry, were small compared to the thickness of the films or of the laminar structure. A depth of approximately 6 µm was reached when the sputtering time was 250 s, which corresponds to the X-axis in Figure 3 (very long sputtering times, 1600 s, reached to 55 µm).

Particularly, only in the case of S-PLA-3/0 films (Figure 4d), the laminar structure is not observed, probably because the magnesium particles favor a better packing of the polymer chains: the interaction between the Mg^2+^ and the oxygen atoms present in the PLA structure could be stronger than the steric hindrance caused by the methyl groups of the polymer chain. After adding surfactant, the laminar structure is again observed (Figure 4e,f), and comparing the cross-section images of the films containing 10% (*w*/*w*) of CTAB, it seems that, in the case of the Mg-containing films (S-PLA-3/10), the polymer matrix arrangement inside the sheets is slightly more compact than in the case without Mg. This would be in agreement with the fact that the CTAB favors the mixing between the Mg particles and the PLA [10].

## 4. Conclusions

According to surface mass spectra, at CTAB concentrations above 5% (*w*/*w*), there is a noticeable presence of surfactant on the film surface. Moreover, the presence of CTAB in the polymer matrix with and without magnesium improves the wettability of the films, making them more suitable for cell adhesion. In addition, the higher the hydrophilicity, the higher the surfactant concentration, because there is a higher accumulation at the film–substrate interface, as demonstrated by ToF-SIMS surface mass spectra.

A layer-like distribution near the surface is revealed by the depth profiles. Although it depends on the surfactant concentration and the presence of Mg, a CTAB + PLA mixed layer and a surfactant exclusion zone can always be observed.

## Figures and Tables

**Table 1:** Contact angles of water (θ_W_) and diiodomethane (θ_D_) on silicone, glass and the silicone and glass-casted films made of PLA, Mg particles and CTAB at the film-substrate interface.

Sample	θ_W_ (°)	θ_D_ (°)	Sample	θ_W_ (°)	θ_D_ (°)
Silicone	113 ± 2	82 ± 6	Glass	43 ± 4	48 ± 1
S-PLA-0/0	107 ± 1	64 ± 2	G-PLA-0/0	75 ± 4	42 ± 2
S-PLA-0/0.5	106 ± 3	59 ± 1	G-PLA-0/0.5	78 ± 5	45 ± 3
S-PLA-0/1	99 ± 4	55 ± 1	G-PLA-0/1	89 ± 7	50 ± 5
S-PLA-0/5	78 ± 10	70 ± 3	G-PLA-0/5	68 ± 7	50 ± 4
S-PLA-0/10	83 ± 8	74 ± 2	G-PLA-0/10	65 ± 11	50 ± 5
S-PLA-3/0	99 ± 4	57 ± 12	G-PLA-3/0	67 ± 3	46 ± 4
S-PLA-3/0.5	99 ± 3	66 ± 1	G-PLA-3/0.5	69 ± 3	43 ± 2
S-PLA-3/1	88 ± 5	64 ± 1	G-PLA-3/1	67 ± 5	51 ± 5
S-PLA-3/5	66 ± 1	60 ± 1	G-PLA-3/5	54 ± 3	58 ± 7
S-PLA-3/10	61 ± 2	65 ± 1	G-PLA-3/10	34 ± 2	51 ± 1

**Table 2:** The dispersive (γd) and polar (γp) components and the surface tension of the solid (γS), calculated using the Fowkes model.

Sample	γ^d^ (mJ/m^2^)	γ^p^ (mJ/m^2^)	γ_s_ (mJ/m^2^)	Sample	γ^d^ (mJ/m^2^)	γ^p^ (mJ/m^2^)	γ_s_ (mJ/m^2^)
S-PLA-0/0	26 ± 1	0 ± 0	26 ± 1	G-PLA-0/0	38 ± 1	6 ± 2	44 ± 3
S-PLA-0/0.5	29 ± 1	0 ± 0	29 ± 1	G-PLA-0/0.5	37 ± 2	5 ± 2	42 ± 4
S-PLA-0/1	31 ± 1	0 ± 1	32 ± 1	G-PLA-0/1	34 ± 3	2 ± 2	36 ± 5
S-PLA-0/5	23 ± 2	9 ± 6	32 ± 7	G-PLA-0/5	34 ± 2	10 ± 4	45 ± 7
S-PLA-0/10	21 ± 1	8 ± 5	28 ± 6	G-PLA-0/10	34 ± 3	12 ± 7	46 ± 10
S-PLA-3/0	30 ± 7	0 ± 1	31 ± 8	G-PLA-3/0	36 ± 2	10 ± 2	46 ± 5
S-PLA-3/0.5	25 ± 1	1 ± 1	26 ± 1	G-PLA-3/0.5	38 ± 1	8 ± 2	46 ± 3
S-PLA-3/1	26 ± 1	4 ± 2	30 ± 2	G-PLA-3/1	33 ± 3	11 ± 4	45 ± 6
S-PLA-3/5	29 ± 1	14 ± 1	42 ± 1	G-PLA-3/5	30 ± 4	21 ± 4	50 ± 8
S-PLA-3/10	26 ± 0	18 ± 2	44 ± 2	G-PLA-3/10	34 ± 1	31 ± 2	64 ± 3

## Data Availability

Data is contained within the article or Supplementary Materials.

## Supplementary Materials

The following are available online: Figure S1. Relative intensities of Br^−^ and C_4_H_7_O_2_^−^ ions as a function of sample type for PLA films containing CTAB and Mg casted on silicone and glass (side exposed to air); Figure S2. Depth profiles of G-PLA-0/0 (a), G-PLA-0/1 (b), G-PLA-0/10 (c), G-PLA-3/0 (d), G-PLA-3/1 (e) and G-PLA-3/10 (f) films; Figure S3. Depth profiles of S-PLA-0/0 (a), S-PLA-0/1 (b), S-PLA-0/10 (c), S-PLA-3/0 (d), S-PLA-3/1 (e) and S-PLA-3/10 (f) films (side exposed to air); Figure S4. Depth profiles of G-PLA-0/0 (a), G-PLA-0/1 (b), G-PLA-0/10 (c), G-PLA-3/0 (d), G-PLA-3/1 (e) and G-PLA-3/10 (f) films (side exposed to air); Figure S5. Cross-section images of G-PLA-0,1 (a), G-PLA-0,10 (b), G-PLA-3,1 (c) and G-PLA-3/10 (d) films.

## Sample Availability

Sample of the compounds are not available from the authors.
