# Supplementary material for: Characterization of Magnesium-Polylactic Acid Films Casted on Different Substrates and Doped with Diverse Amounts of CTAB
Source: Molecules. 2021 Aug 9;26(16):4811. doi: 10.3390/molecules26164811 (PMC8398461; doi:10.3390/molecules26164811)
Supplement: Supplementary file 1 [file molecules-26-04811-s001.zip › molecules-1316421-supplementary.pdf]

Characterization of magnesium-polylactic acid films casted on different substrates and doped with diverse amounts of CTAB

Margarita Hierro-Oliva, Verónica Luque-Agudo, Amparo M. Gallardo-Moreno, M. Luisa González-Martín*

Department of Applied Physics, University of Extremadura, Badajoz, Spain; Networking Research Center on Bioengineering, Biomaterials and Nanomedicine (CIBER-BBN), Badajoz, Spain; University Institute of Extremadura Sanity Research (INUBE), Badajoz, Spain.

*Corresponding author: mlglez@unex.es

SUPPORTING INFORMATION

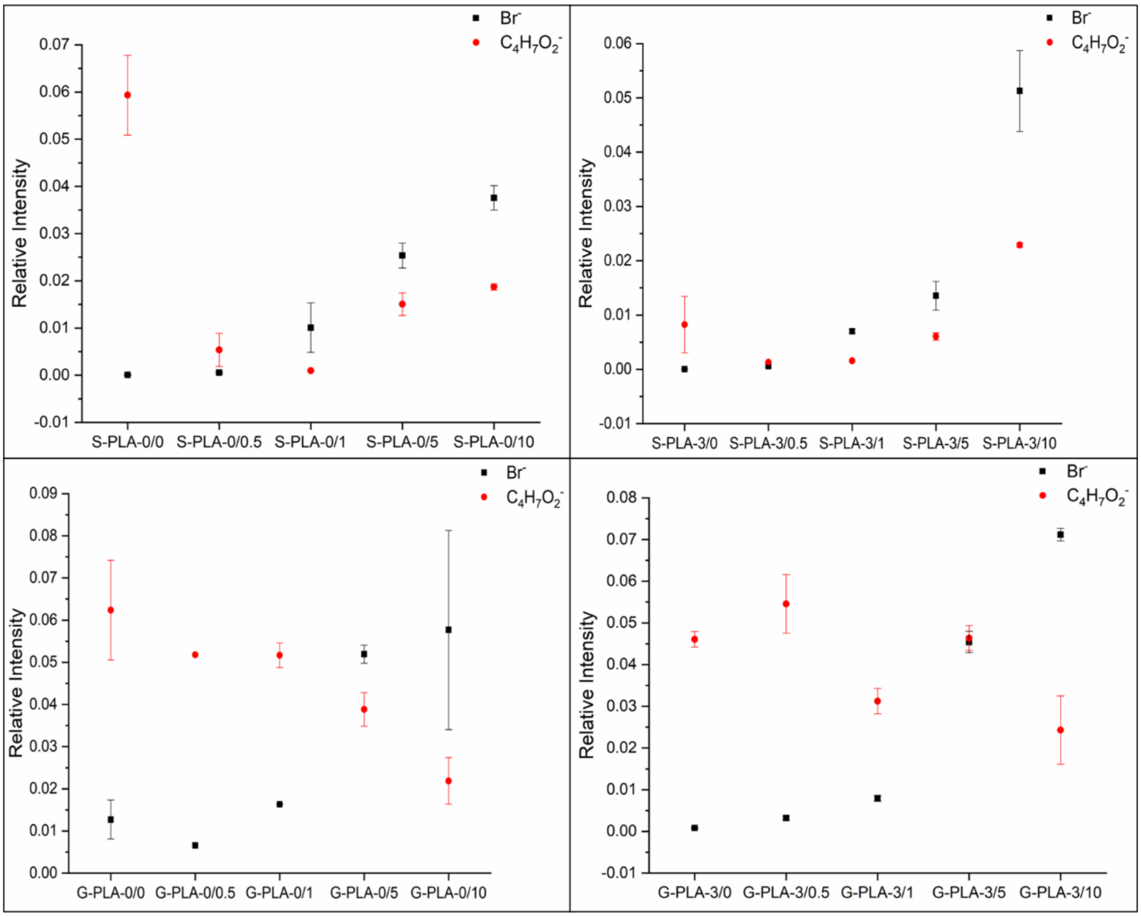

Figure S1. *Relative intensities of Br^- and $C_4H_7O_2^-$ ions as a function of sample type for PLA films containing CTAB and Mg casted on silicone and glass (side exposed to air).*

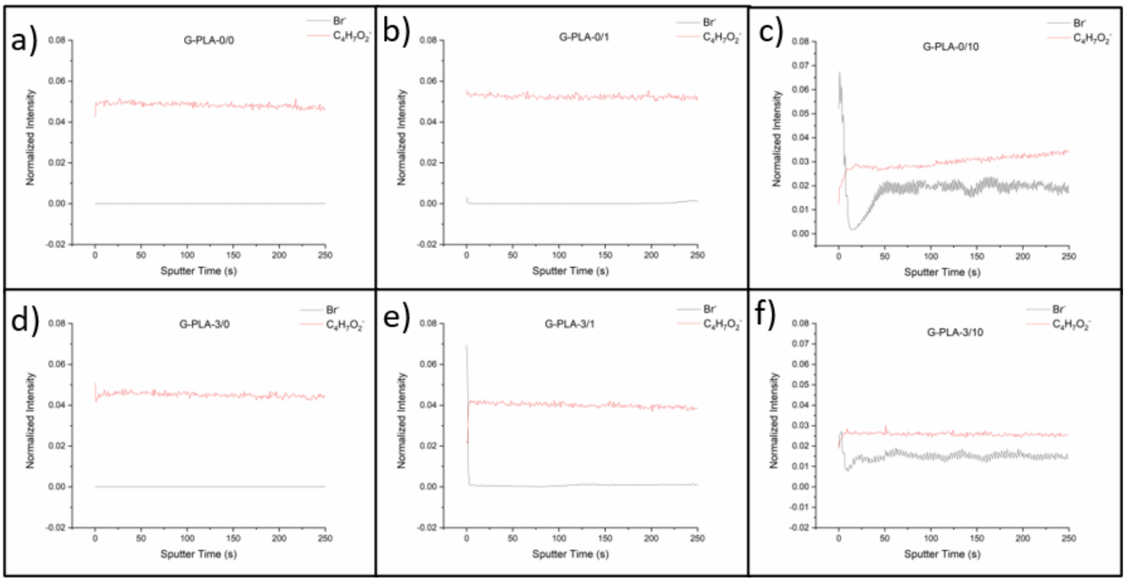

Figure S2. *Depth profiles of G-PLA-0/0 (a), G-PLA-0/1 (b), G-PLA-0/10 (c), G-PLA-3/0 (d), G-PLA-3/1 (e) and G-PLA-3/10 (f) films.*

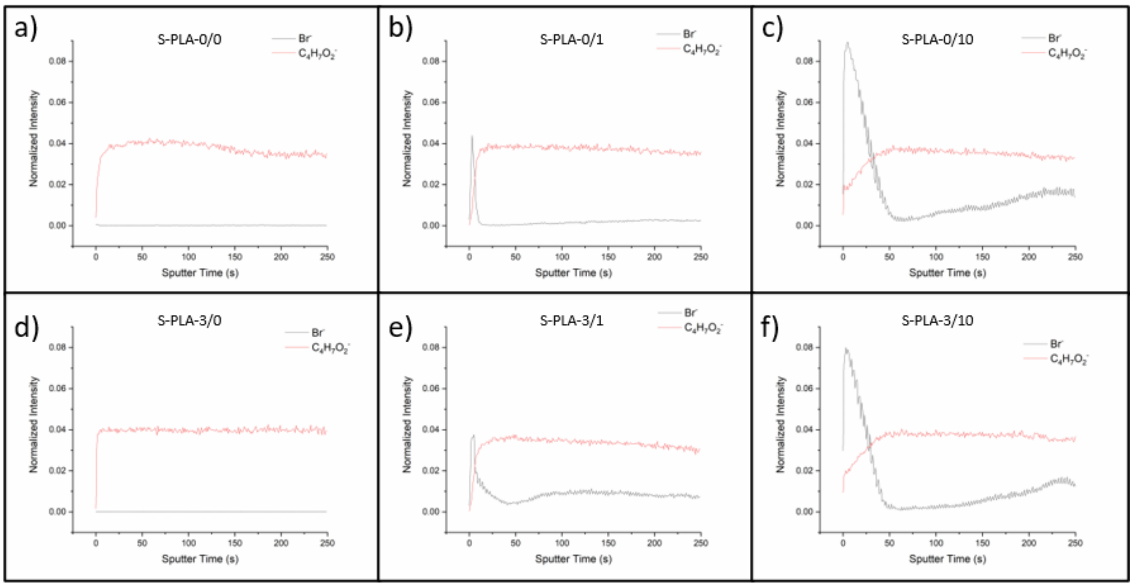

Figure S3. *Depth profiles of S-PLA-0/0 (a), S-PLA-0/1 (b), S-PLA-0/10 (c), S-PLA-3/0 (d), S-PLA-3/1 (e) and S-PLA-3/10 (f) films (side exposed to air).*

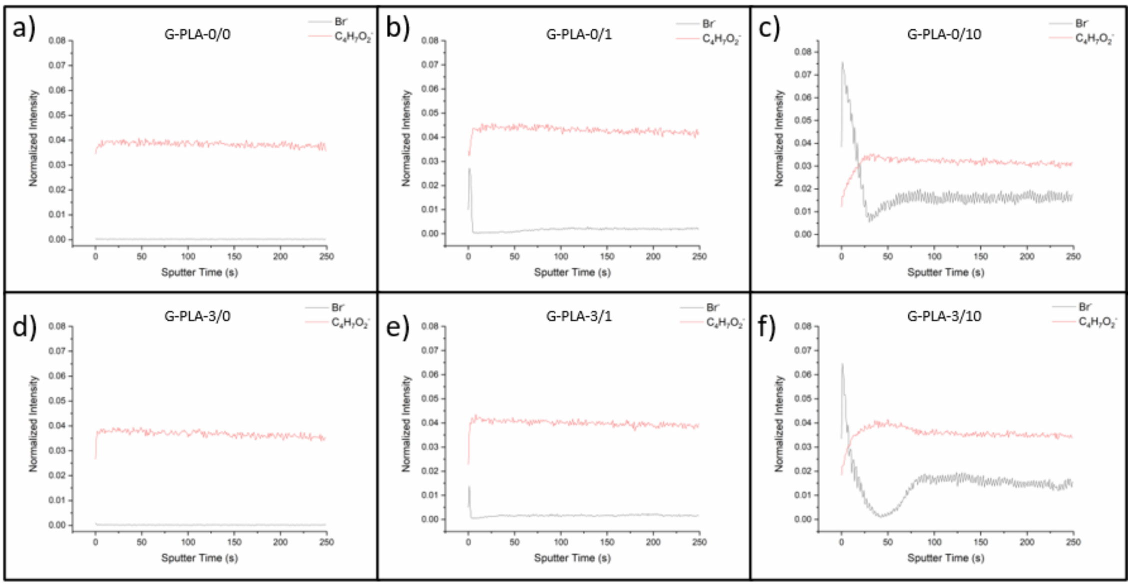

Figure S4. *Depth profiles of G-PLA-0/0 (a), G-PLA-0/1 (b), G-PLA-0/10 (c), G-PLA-3/0 (d), G-PLA-3/1 (e) and G-PLA-3/10 (f) films (side exposed to air).*

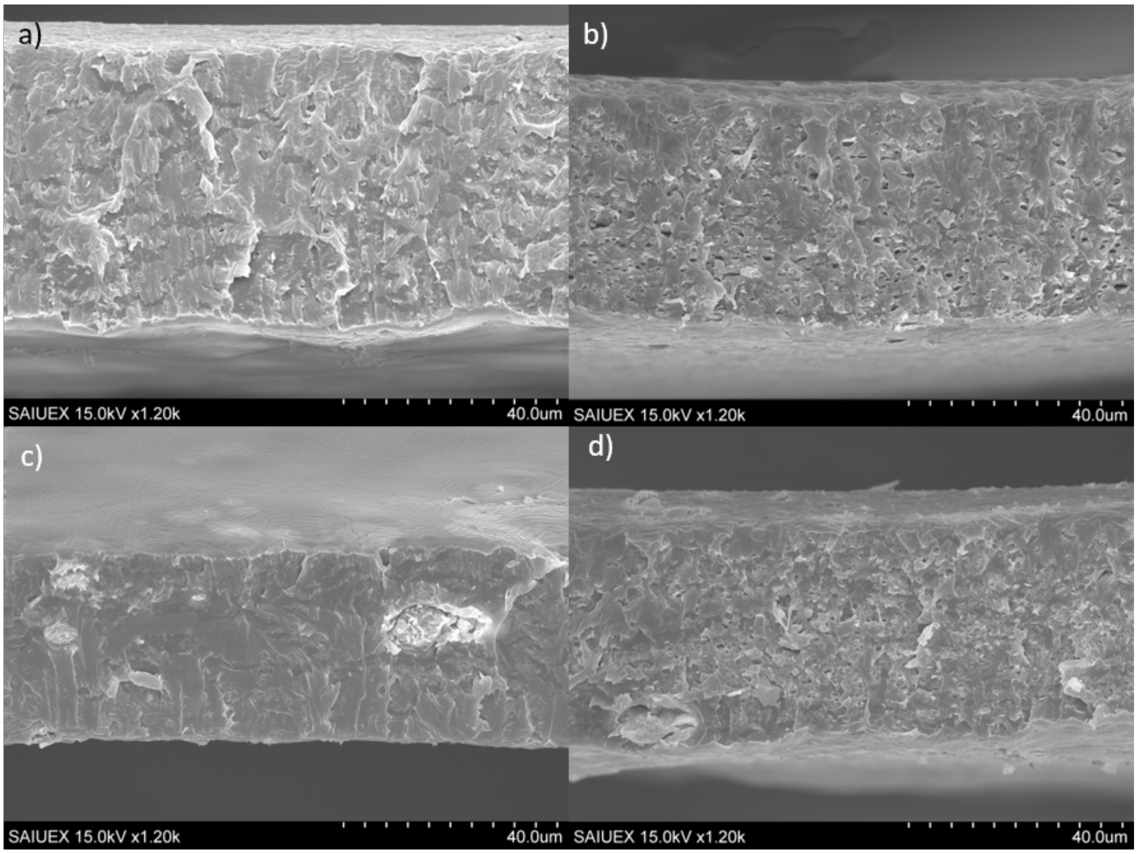

Figure S5. *Cross-section images of G-PLA-0,1 (a), G-PLA-0,10 (b), G-PLA-3,1 (c) and G-PLA-3/10 (d) films.*
